# Thermal Analysis in the Diagnostics of Inflammatory and Septic Conditions

**DOI:** 10.3390/biomedicines14102290

**Published:** 2026-10-09

**Authors:** Dénes Lőrinczy, László Bogdán, Árpád Pillér, Árpád Dandé, László G. Nöt

**Affiliations:** 1 Department of Biophysics, School of Medicine, University of Pécs, Szigeti Str. 12, H-7624 Pecs, Hungary; 2 Trauma Unit, Center for Musculoskeletal Surgery, ‘Kaposi Mór’ Teaching Hospital of Somogy County, Tallián Gy. u. 20–32, H-7400 Kaposvar, Hungary; 3 Department of Traumatology and Hand Surgery, School of Medicine, University of Pécs, Szigeti Str. 12, H-7624 Pecs, Hungary; 4 Institute of Physiotherapy and Sport Science, Faculty of Health Sciences, University of Pécs, 4 Vörösmarty Street, H-7621 Pecs, Hungary

**Keywords:** fracture related infection (FRI), periprosthetic joint infection (PJI), thermal liquid biopsy (TLB), isothermal calorimetry (IMC), differential scanning calorimetry (DSC), inflammatory, septic

## Abstract

**Background/Objectives**: Differentiating between inflammatory and septic purulent processes in the human body remains difficult today. A number of research techniques have emerged recently, and calorimetric investigations have shown promise in the study of malignancies and inflammatory and septic diseases. The purpose of our review article is to present and summarize the potential applications of calorimetric measurements in the study of human inflammatory and septic disorders. **Methods**: For calorimetric measurements, a small volume of liquid (blood plasma, synovial fluid) or tissue sample is sufficient. The thermal data and denaturation curve obtained during the calorimetric procedure performed by a differential scanning calorimeter (DSC) show the structural changes in the proteins of the examined sample. The thermograms and the calculated thermal values (∆*H_cal_*, calorimetric enthalpy, *T_m_* melting or denaturation temperature) can demonstrate the unique variation and characteristics of the studied pathologies. These calorimetric procedures have recently been referred to as thermal liquid biopsy (TLB). **Results**: With the help of proliferation thermograms provided by isothermal calorimetric measurements (IMCs), it is also possible to detect bacteria present in low concentrations in different samples. Based on the literature and on our workgroup’s data, calorimetric analyses have been successfully applied in the differential diagnosis of numerous human inflammatory diseases (psoriasis, systemic lupus erythematosus, synovitis) and septic conditions (septic arthritis, periprosthetic joint infection, fracture related joint infection). **Conclusions**: Considering the advantages of the technique, calorimetric analyses could serve as a complementary method in the clinical diagnostics of septic conditions in the future.

## 1. Introduction

Despite the development of laboratory diagnostics and medical imaging, the clinical investigation of septic joint and bone processes is still challenging. Diagnostic criteria of bacterial joint infections have been widely investigated during recent years.

Since bacterial, viral and fungal infections can all cause septic arthritis, the source and type of microorganism resulting in joint infection should be identified for successful treatment. Pathomechanisms of septic arthritis can be hematogenous spread, direct entry or contagious spread, originating from adjacent osteomyelitis [1]. The presence of previously inserted medical implants can increase the risk for septic infection. The diagnostic criteria published in different guidelines involve both histological examination of involved tissue and microbiological culturing, as a ‘gold standard’ for identification of pathogen bacteria [1].

Recently, new, consensus-based guidelines have been published for the treatment of fracture-related infections (FRIs) [1,2,3,4]. An international expert group defined the basic criteria for the diagnosis of FRIs [5]. Similarly, to the FRI guidelines, diagnostic and treatment protocols for periprosthetic infections (PJIs) have also been published recently [6,7,8].

The treatment protocols of both FRIs and PJIs require the identification of pathogens and suggest early, specific antibiotic treatments, based on the microbiological examinations and antibiotic resistance tests [1,6,7,8,9].

While histological examination and microbiological culturing provide the most valuable information about the presence of neutrophil cells and the type and resistance of bacteria, the time required for these examinations is from 2 days to several weeks.

Meanwhile, Nynatten et al. studied plasma proteomics to find novel biomarkers of sepsis in critically ill patients [10]. Blood was collected from septic patients at ICU Day 1 and Day 3. Using proximal extension assays (PEAs), a total of 1161 plasma proteins were analyzed. The authors found correlations between protein expression and clinical/laboratory variables.

Another possibility is to monitor the increased temperature in an area suspected of septic arthritis, using thermal imaging, compared to the opposite-side joint. Involving 15 patients with septic arthritis and 34 patients with non-septic arthritis, Gunay et al. found a much higher difference in the temperatures in the septic group, compared with the non-septic group [11].

Traditional biomarkers such as C-reactive protein (CRP) and procalcitonin (PCT) are elevated in septic infections. However, CRP is known to be elevated in other inflammatory states, and it has a low specificity to diagnose sepsis. PCT can be used as an indicator for antibiotic treatment and increased early levels of PCT usually indicate unfavorable prognosis. CRP and PCT have limited ability to predict the outcome and mortality of disease in septic shock [12,13,14,15]. Therefore, there is a need for new investigation methods in the differential diagnostic procedure of septic inflammations.

In recent years, the effectiveness of calorimetric measurements in diagnostics procedures has been proven in numerous scientific and clinical studies [16,17,18,19,20,21,22,23,24,25]. Isothermal calorimetry (IMC) is able to determine the rate of growth of pathogens in different samples [26]. The differential scanning calorimetry (DSC) is also a widely used method for studying the structural modifications of proteins in different kinds of inflammatory diseases [27,28,29,30]. The thermal analysis of plasma proteins has been a reliable tool in the diagnosis and the follow-up monitoring of various inflammatory and malignant diseases.

Taken together, the purpose of our review article was to demonstrate the application of thermal analyses for the investigation of septic and non-septic inflammatory conditions.

## 2. Isothermal Microcalorimetry (IMC) and Differential Scanning Calorimetry (DSC)

For calorimetric analysis of the sample types discussed in our review, two types of methods can be highlighted among the available techniques: isothermal microcalorimetry (IMC) and differential scanning calorimetry (DSC). The IMC measures heat produced by viable microorganisms, whereas DSC mainly reflects thermal changes in proteins and other macromolecules. Notably, these instruments are capable of differential scanning calorimetric analysis and can function in isothermal mode, too (isoperibol calorimetry), meaning the DSC instrument can function as an IMC instrument, measuring heat production of bacteria.

**Isothermal microcalorimetry (IMC):** 

Isothermal microcalorimetry detects the heat flow produced or absorbed over time, at constant temperature. During analysis, heat production of the test samples is always compared to a standardized reference sample. With the help of IMCs, thermodynamics, kinetics and metabolic processes can be monitored, including bacterial growth [25,31,32].

**Figure 1:**
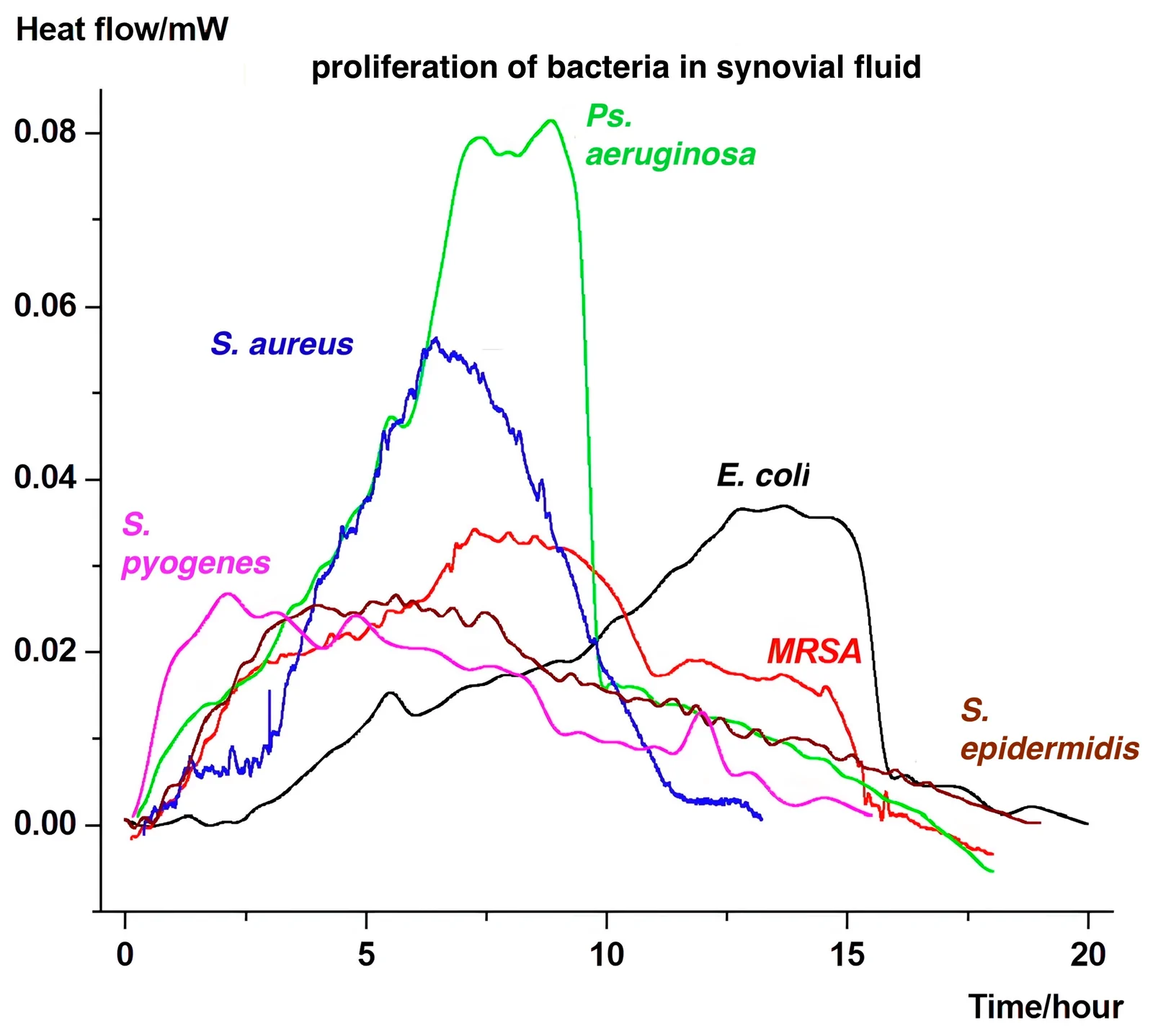
**Proliferation thermograms of different bacterial strains:** *E. coli* (*Escherichia coli*), MRSA (Methicillin-resistant *Staphylococcus aureus*), *Ps. aeruginosa* (*Pseudomonas aeruginosa*), *S. aureus* (*Staphylococcus aureus*), *S. epidermidis* (*Staphylococcus epidermis*), and *S. pyogenes* (*Streptococcus pyogenes*). Concentration: 10^3^ CFU/mL. The vertical axis indicates the heat flow (in mW) and the horizontal axis shows the time (hour). Samples were run in technical triplicate, and each curve represents the average of the three measurements [23].

The output parameters of IMC are thermograms, indicating the heat flow as a function of time (*dq*/*dt*). The main calculated output parameters include the calorimetric enthalpy change (∆*H_cal_*) (area under the curve) and calculation of different time-points of the proliferation thermograms, such as the starting (*t_s_*) and endpoint (*t_e_*) as well as the maxima (*t_m_*) of proliferation. As an example, for differential scanning calorimeters (DSCs) in isotherm mode (isoperibol), Dandé et al. inoculated different types of bacterial strains with a final concentration of 10^3^ or 10^5^ CFU/mL to human synovial samples after reaching the thermal equilibrium (SETARAM Micro DSC-II, 7 rue de l’Oratoire 69300 Caluire-et -Cuire Lyon, France). In isothermal mode, the proliferation thermograms were detected and thermal parameters calculated (Figure 1). The thermograms show different, individual patterns, referring to different types of bacterial strains [33].

**Figure 2:**
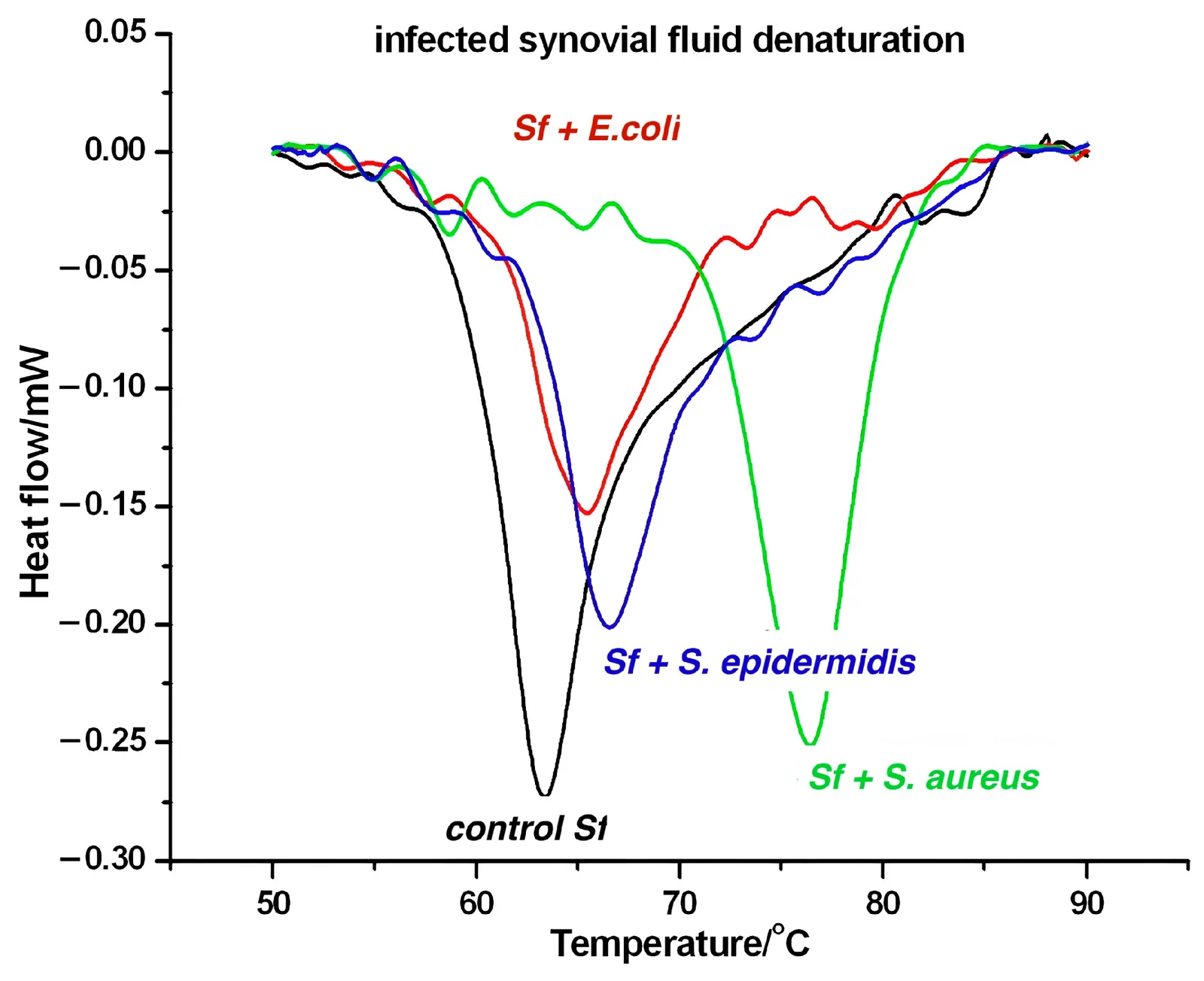
**Thermal denaturation scans of synovial fluid (Sf) samples infected with different bacterial strains.** *E. coli* (*Escherichia coli*), *S. aureus* (*Staphylococcus aureus*) and *S. epidermidis* (*Staphylococcus epidermidis*). Concentration: 10^3^ CFU/mL. The vertical axis indicates the heat flow (in mW) and the horizontal axis shows the temperature (°C). Endothermic reaction is directed downwards. Samples were run in technical triplicates, and each curve represents the average of the three measurements [23].

The differential scanning calorimeter (DSC) in ‘scanning mode’ detects the difference heat flow between the examined test sample and the reference sample. During the measurements, the sample undergoes phase transitions (‘melting’) due to heating at a standard rate and releasing or absorbing heat (endothermic or exothermic reactions) compared to the reference (SETARAM Micro DSC-II, 7 rue de l’Oratoire 69300 Caluire-et -Cuire Lyon, France). The denaturation scans show the heat flow in the function of temperature or time. The main output parameters can be calculated from the DSC scans: enthalpy of transitions (∆*H_cal_*, calorimetric enthalpy refers to each transition) and *T_m_* (melting or denaturation temperature), indicating a peak temperature, when the phase transition (‘melting’) occurs at least in 50%, and its *T*_½_ shows the half temperature width of denaturation heat (it shows the direction of structural change: at low values the structure becomes more ordered; the higher one refers to the loosening of internal structure of macromolecules). The results are normalized to the total (dry or wet) sample mass (Figure 2).

In the case of studying structural changes in samples containing proteins, for example, different types of collagens [34,35], the thermal parameters produced by DSC reflect the thermal (structural) stability of the protein components.

**Figure 3:**
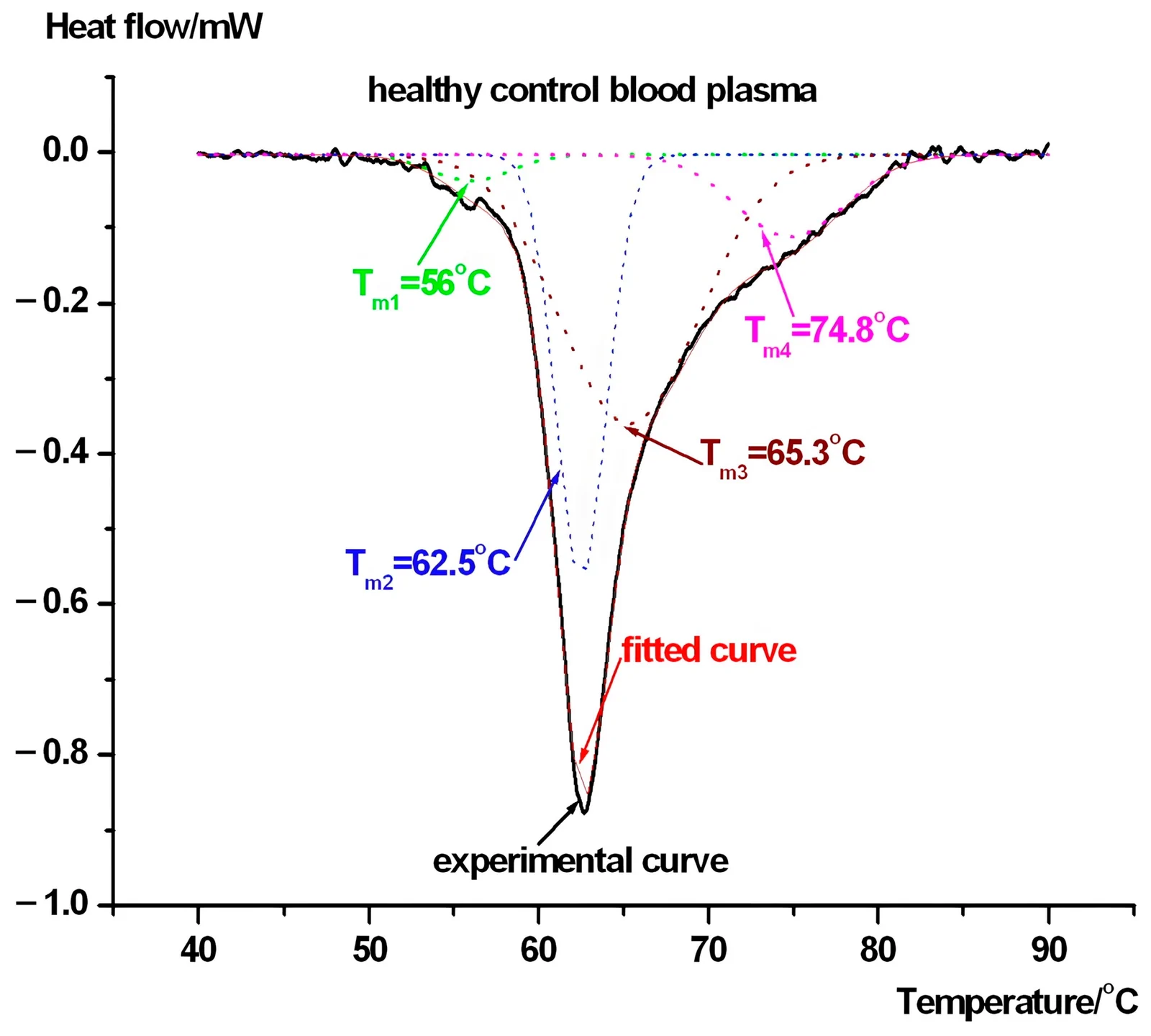
**Deconvoluted thermal denaturation scan of plasma sample of a healthy patient.** The different peaks (*T_m_*_1_–*T_m_*_4_) indicate the thermal transition of different plasma protein components. The experimental curve means the original denaturation and the fitted curve represents the area of the sum of the peaks achieved by deconvolution [36]. The vertical axis indicates the heat flow (in mW) and the horizontal axis shows the temperature (°C).

Using deconvolution of denaturation scans, the area under the curve can be divided into the sum of sub-areas that are nearly equal to the area of the original curve (SETARAM Micro DSC-II, 7 rue de l’Oratoire 69300 Caluire-et -Cuire Lyon, France). Since macromolecule solutions consist of several components, if the denaturation parameters of each component are determined previously, the deconvolution technique allows them to follow the thermal effect caused by an intervention by dividing the experimental curve into components, using their denaturation temperature. The maximum of the deconvolved curve is around the original denaturation temperature and varies until the best curve fit is achieved (Figure 3).

If the *T_m_* is greater than the original component, then the intervention has stabilized the evaluated structural unit. If it is smaller, then the relationship between the components weakens. The change in the area under the curve indicates the contribution of the given structural unit to the total denaturation enthalpy: in case it is increased, the structure becomes more stable; if it is decreased, the structure becomes thermodynamically less stable.

In another set of experiments, synovial samples infected with different bacterial strains were subjected to differential scanning calorimetric analyses in ‘scanning mode’. The denaturation curves show the changes in the thermal characteristics of synovial fluids due to the presence of bacteria. The changes in calorimetric enthalpy and the increased melting temperature mean that the protein content of synovial fluid becomes more ‘rigid’ in response to bacterial infection [33,37,38].

## 3. Application of Thermal Analysis in the Diagnostics of Inflammatory Conditions

In inflammatory diseases, DSC can also offer a ‘fingerprint’ of the serum proteome, identify structural alterations and shift in protein composition (such as immunoglobulins and acute-phase proteins).

Psoriasis is a chronic, auto-inflammatory skin disease. Moezzi et al. demonstrated in a preliminary study that the thermal changes (melting temperature (*T_m_*) and calorimetric enthalpy (∆*H_cal_*) of blood plasma of psoriatic patients correlate with the PASI (psoriasis area severity index) and the efficacy of therapy (Table 1). In the study of Lőrinczy et al., plasma samples were obtained from psoriatic patients, receiving steroid, retinoid or biological therapy. Using differential scanning calorimetry and the deconvoluted plasma protein profiles, it has been shown that the changes in the individual peak transitions corresponded to the psoriasis stage and different treatments. They concluded that the anti-psoriatic drug therapies can affect the plasma proteins compositions [36,39].

Studying the autoimmune condition of Systemic Lupus Erythematosus (SLE), the plasma thermogram profiles of 300 non-SLE and 300 SLE patients were compared. Garbett et al. have found that median sensitivity, specificity, and overall accuracy of SLE classification based on plasma thermograms was comparable with a classification based on antibody tests [40]. They also demonstrated a greater difference between the thermograms of SLE and non-SLE samples in female patients and in cases of anemia.

Lin et al. have compared the heat denaturation curves (HDCs) and proteomic profiles of serum proteins collected from healthy individuals with patients with joint-inflammatory symptoms (*n* = 50) or specifically with rheumatoid arthritis (*n* = 27), showing significant differences between of the HDCs of the different samples [41].

It is known that aneurysmal subarachnoid hemorrhage (aSAH) can be accompanied by both inflammatory and metabolic responses, affecting the clinical outcome. In a recent publication, serum samples collected of patients 1 day after aSAH were compared to healthy individuals. Severe clinical status was characterized by increased albumin *T_m_*, decreased albumin cooperativity and heat capacity changes (∆*C_p_*). Additionally, their data showed more immunoglobulin-related contributions in the higher temperature range, based on the deconvoluted thermograms [42].

In the diagnostic procedure of multiple sclerosis (MS), thermal liquid biopsy (TLB) of blood plasma samples could provide an insight into the overall real-time changes in MS patients’ plasma proteome. Annesi et al. have demonstrated that TLB had about 70% sensitivity and specificity in the diagnostic process, by comparing thermograms of plasma proteomes 45 healthy and 45 MS patients [43].

In another study, Hermoso-Durán et al. have compared the TLB samples collected from healthy, benign and premalignant cysts patients. They have found that the TLB serum score was able to differentiate between healthy and cysts patients (71% sensitivity and 98% specificity) and also between benign and premalignant cysts (75% sensitivity and 67% specificity) [44].

Differential scanning calorimetry was also found to be useful in the evaluation of orthopedic and rheumatoid inflammatory disease, utilizing small-sized tissue samples [34,35].

Since collagens are one of the most elementary components of musculoskeletal systems, Wiegand et al. have examined extensively the structural changes in collagens, using differential scanning calorimetry [34,35]. Of the 25 different types of collagens, 90% of the collagen is type I, occurring in tendons, ligaments, and bones. Other types, like type II. is found generally in hyaline cartilage and type III in granulation tissues. Therefore, most thermoanalytic results of Wiegand et al. reflect the structural changes in collagen type I.

By comparing the inflamed and intact human tissue samples from individuals with Dupuytren contracture, De Quervain’s tenosynovitis, and carpal tunnel syndrome (CTS), they have shown a marked increase in thermal enthalpy. Of the assessed samples, carpal tunnel syndrome (∆*H_cal_* healthy: 4.0 Jg^−1^ vs. inflamed: 5.9 Jg^−1^, *p* < 0.05) and De Quervain’s tenosynovitis (∆*H_cal_* healthy: 3.84 Jg^−1^ vs. inflamed: 5.39 Jg^−1^, *p* < 0.05) were accompanied with the greatest increase in thermal enthalpy. In addition, they found a non-significant increase in the denaturation temperature in the inflamed tissue samples, compared to the healthy samples [34,35].

Based on their studies, Wiegand et al. explained their findings that chronic inflammation, as a pathological background for these diseases, could have an impact on regulatory pathways that cause fibroblast proliferation and interfere with the formation of the extracellular matrix. The observed alterations in the thermal properties may be explained by the formation of irregular bundles and variations in the balance of various collagens [34,35].

During hip arthroplasties, the joint capsule is partially or fully resected. After surgery, a new ‘pseudo-capsule’ develops. Under certain conditions, such as adverse local tissue reaction (ALTR) or septic infection, this pseudomembrane layer can be affected by inflammatory reactions. Bűcs et al. have collected small-sized samples (4  ×  4  ×  20 mm) from the anterior part of the pseudo-capsule during secondary hip arthroplasties. The indication of the secondary surgery was aseptic loosening, septic loosening, or condition after antibiotic-impregnated spacer implantation. They found that the denaturation temperature and thermal enthalpy correlate with the severity of inflammation. Compared to aseptic loosening, the pseudomembrane collected from patients with antibiotic-impregnated spacers showed a 20% decrease in thermal enthalpy, while the septic loosening resulted in an about ~ 50% decrease. The authors claimed that it is due to the enhanced structural loosening of pseudo-capsules [45].

By evaluating the interfacial membrane collected from patients after failed hip arthroplasties, Naumov et al. have shown that calorimetric analyses could help in the differential diagnostics of interfacial membranes. These membranes develop between the surface of the bone and the implant or bone cement, formed of granulation tissue. The aseptic membrane exhibited a higher denaturation temperature and lower thermal enthalpy, compared to the infected membranes (*T_m_* = 62.2 °C, ∆*H_cal_* = 2.13 Jg^−1^ and *T_m_* = 60.2 °C and ∆*H_cal_* = 3.22 Jg^−1^, respectively) [46].

**Table 1:** Application of thermal analysis in the diagnostics of inflammatory conditions.

Disease/Tested Samples	Method	Publications	Result Summary
Psoriasis, blood plasma of psoriatic patients	Differential scanning calorimetry (DSC)	Moezzi et al. Lőrinczy et al. [36,39]	Thermal changes in plasma proteins correlated with the PASI (psoriasis area severity index) and with the efficacy of therapy; anti-psoriatic drug therapies affected the plasma proteins compositions
Systemic Lupus Erythematosus (SLE), plasma of non-SLE and SLE patients	Differential scanning calorimetry (DSC)	Garbett et al. [40]	Plasma thermogram profiles of 300 non-SLE and 300 SLE patients were compared: median sensitivity, specificity, and overall accuracy of SLE classification based on plasma thermograms was comparable with a classification based on antibody tests
Rheumatoid arthritis, human serum albumin	Differential scanning calorimetry (DSC)	Lin et al. [41]	Comparing heat denaturation curves and proteomic profiles of healthy individuals with patients with joint-inflammatory symptoms (*n* = 50) or specifically with rheumatoid arthritis (*n* = 27)
Aneurysmal subarachnoid hemorrhage (aSAH), serum samples	Differential scanning calorimetry (DSC)	Lőrinczy and Csécsei [42]	Severe clinical status was characterized by increased albumin *T_m_*, decreased albumin cooperativity and heat capacity changes (∆*C_p_*)
Multiple sclerosis (MS), thermal liquid biopsy (TLB) of blood plasma samples	Differential scanning calorimetry (DSC)	Annesi et al. [43]	TLB had about 70% sensitivity and specificity in the diagnostic process, by comparing thermograms of plasma proteomes 45 healthy and 45 MS patients
Benign and premalignant pancreatic cyst diagnosis, TLB	Differential scanning calorimetry (DSC)	Hermoso-Durán et al. [44]	TLB serum score discriminated between healthy and cyst patients (71% sensitivity and 98% specificity) and between benign and premalignant cysts (75% sensitivity and 67% specificity)
De Quervain’s tenosynovitis, and carpal tunnel syndrome (CTS), tissue samples	Differential scanning calorimetry (DSC)	Wiegand et al. [34,35]	Demonstrated marked increase in thermal enthalpy of inflamed samples: CTS: ∆*H_cal_* healthy: 4.0 Jg^−1^ vs. inflamed: 5.9 Jg^−1^, *p* < 0.05 and De Quervain’s tenosynovitis: ∆*H_cal_* healthy: 3.84 Jg^−1^ vs. inflamed: 5.39 Jg^−1^, *p* < 0.05
Revision hip arthroplasty, samples collected from hip pseudomembrane	Differential scanning calorimetry (DSC)	Bűcs et al. [45]	Compared to aseptic loosening, the pseudomembrane collected from patients with antibiotic-impregnated spacer showed a 20% decrease in thermal enthalpy, while the septic loosening resulted in an about ~ 50% decrease.
Failed hip arthroplasties, tissue samples of interfacial membrane	Differential scanning calorimetry (DSC)	Naumov et al. [46].	The aseptic membrane exhibited a higher denaturation temperature and lower thermal enthalpy, compared to the infected membranes (*T_m_* = 62.2 °C, ∆*H_cal_* = 2.13 Jg^−1^ and *T_m_* = 60.2 °C and ∆*H_cal_* = 3.22 Jg^−1^, respectively)

## 4. Application of Thermal Analysis in the Diagnostics of Septic Conditions

Starting with the most fundamental microbiological examinations, DSC were used to follow growth of *E. coli* bacteria. The gold-standard method to measure bacterial growth is the plate-count, which takes 24–48 h or turbidimetry, which cannot be used in opaque environment. As Rangel-Vargas et al. reported, during the initial growth phase, bacteria released energy [47]. The thermograms produced by their measurements correlated to the cell growth and activity, supported by plate counts. The differential scanning calorimetry made it possible to follow the real-time growth of *E. coli*, between few minutes up to 5 h (Table 2).

In addition, Muntean et al. used microcalorimetry (micro-DSC) to discriminate between bacterial strains, based on their thermograms [48]. The thermograms were recorded from broth cultures of *Escherichia coli* (ATCC 25922) and *Staphylococcus aureus* (ATCC 25923) at an initial 1/100 dilution of 0.1 McFarland suspension. They claimed that the thermograms behaved as a kinetic fingerprint; *E. coli* showing a faster growth and more intense kinetics.

Venous catheter infections can cause severe complications, such as bloodstream infections. Austin et al. collected wild-type bacteria (*Escherichia coli*, *Staphylococcus epidermidis*, *Klebsiella pneumoniae*, and *Enterococcus faecium*) from blood cultures of adult inpatients receiving parenteral nutrition. Isothermal microcalorimetry confirmed the bacterial infection in all cases and thermograms showed different patterns, based on the bacterium species. Authors found their data highly reproducible within each species. In case of *S. epidermidis* and *K. pneumoniae*, the antibiotic resistances were also tested [49].

Based on the thermal parameters and DSC scans, characteristic thermal ‘fingerprints’ of different bacterial strains can generate, mostly evaluated the heat denaturation of cellular components like proteins, ribosomes, and lipids, typically between 40 °C and 130 °C. For example, Braissant et al. have successfully utilized micro-calorimetry in the assessment of bacterial growth in artificial urine samples inoculated with the most common UTI pathogens: *Escherichia coli*, *Enterococcus faecalis* and *Enterococcus faecium*). They concluded that isothermal microcalorimetry (IMC) was capable to evaluate the antibiotic resistance within 7 h with a sensitivity of 95% and specificity of 91%, using Vitek-2 system (bioMérieux, Marcy l’Etoile, France) as a reference. The concentration of bacteria used for their experiments was lower (∼10^5^ CFU/mL), compared to concentrations in severe infections (∼10^8^ CFU/mL). Finally, the cost of their measurements was also reported, seeming reasonable vs. other methods [50].

Bonkat et al. have also applied microcalorimetry in clinical urological settings. Briefly, sterile urine samples were inoculated with four different kinds of bacterial strains: *Escherichia coli*, *Proteus mirabilis*, *Enterococcus faecalis* and *Staphylococcus aureus*. At 37 °C, metabolic heat flow (µW = µJ/s) was measured as a function of time. IMC indicated bacterial growth after 3.1 to 17.1 h. Important to note that this time frame is much shorter than the time needed for bacterial culturing. Moreover, the detection limit was less than 10^3^ CFU (colony forming unit) and the heat flow curves showed individual patterns for each bacteria strain, regardless of the starting concentration [51].

Meanwhile, Dandé et al. have studied bacterial infection of human synovial samples. In their first set of experiments, collected synovial samples of patients of different stages of osteoarthritis [33,37,38,52]. Then, samples analyzed with differential scanning calorimetry and they found marked correlation between the degree of osteoarthritis and thermal parameters. The result of DSC analyzes were compared to the Kellgren–Lawrence and modified Kellgren–Lawrence radiological classification systems [53]. It needs to be noted, that only a small, 2–5 mL of the SF was drained by the physician under sterile conditions for these studies. Parallel with the progress of osteoarthritis (OA), a significant increase was observed in the *T*_½_ values (temperature interval of transition temperature at half of the heat flow), which could be an indicator of the loss of cooperativity of the certain thermal domains of the arthritic sample. Due to the progress in the OA, the structure becomes less organized. Similar trends were observed in the changes in thermal enthalpy.

In their second set of experiments, synovial samples have been over infected by different strains of bacteria. Aseptic human SF samples were inoculated with the most common bacterial strains (*Staphylococcus aureus*, *MRSA* (methicillin-resistant *Staphylococcus aureus*), *Staphylococcus epidermidis*, *Streptococcus pyogenes*, *Escherichia coli* and *Pseudomonas aeruginosa*, with colony-forming units (CFU) 10^3^ or 10^5^ colonies/mL broth. Then, isothermal (isoperibol) calorimetric experiments were performed to monitor bacterial growth. Authors have demonstrated that thermal scans were dependent on bacterial concentration. Moreover, the scans showed a unique pattern as a characteristic of different bacterial strains.

Isolation of biofilm forming bacteria attached to implant surfaces has become in the focus of orthopedics research recently. One method is called sonication, when the removed, potentially infected implant is immersed in fluid and subjected to ultrasound waves to de-attach bacteria. Trampuz et al. successfully combined sonication with microcalorimetric measurements. They demonstrated that the sensitivity and specificity of microcalorimetry of sonication fluid were 100% and 97%, respectively. The measurements were all confirmed by standard tissue culturing techniques. The mean time of detection was 10.9 h [54].

Recently, more scientific papers have been published about the use of thermal analysis in the diagnostics of PJI (periprosthetic joint infections) [55]. Morgenstern et al. collected synovial samples by aspiration prior to revision of hip and knee surgery. In 98 of 107 patients (92%), synovial fluid culture and microcalorimetry indicated concordant results (38 PJI and 60 aseptic cases). In addition, they reported statistically significant connection between the leukocyte count of synovial fluid and heat production. The performed linear regression test demonstrated that a 1% increase in leukocyte count was related to a 0.72% increase in peak heat production [22]. Following the PJI/FRI diagnostic guidelines, regularly 3–5 tissue samples are collected during revision surgery to confirm low-grade infection by microbiological culturing and histological examinations. Notably, isothermal microcalorimetry (IMC) has been found useful not only for synovial samples but for tissue evaluation as well.

De Bleeckere et al. investigated the efficacy of isothermal microcalorimetry in the diagnostics of suspected periprosthetic joint infections [56]. In their study, the conventional culturing (CC) technique was compared to culturing in synthetic synovial fluid (SSF2) and using isothermal microcalorimetry (IMC). Culturing in SSF2 showed the highest positivity rate (53.2 %), followed by IMC and CC (34.2 % and 32.9 %, respectively). With the use of IMC, they could rapidly detect the microbial activity in the sample (median detection times between 15.9 h and 15.6 h depending on different culturing media).

In a retrospective study involving large and small joint infections, Dandé et al. have assessed the efficacy of standard microbiological culturing of joint infections, including PJI cases [57]. Data of 72 patients with large joints infections or PJI and 65 patients with small joint infections were evaluated, including inflammatory markers and results of microbiological culturing. Based on the conventional culturing results, the most frequent bacteria strains were the *Staphylococci*: in 43 cases (22.16%) *Staphylococcus aureus*, in 22 cases (11.34%) coagulase-negative *Staphylococcus*, in 3 cases (1.54%) *Staphylococcus epidermidis* and in 4 cases (2.06%) methicillin-resistant *Staphylococcus aureus* (MRSA). Authors concluded that in 30 cases (21.88%), the standard microbiological culturing was not sufficient to diagnose the presence of bacteria.

Meanwhile, Chicos et al. collected tissue samples of patients, underwent revision hip or knee arthroplasties. Their study involved 152 patients with 592 tissue samples. Authors found concordance between standard microbiological culturing and isothermal microcalorimetry (IMC) of 90%. Moreover, comparing the reliability of the two methods, IMC demonstrated a sensitivity of 83%, specificity of 100%, negative predictive value (NPV) of 89%, and positive predictive value (PPV) of 100% for PJI vs. standard culturing showed 69% sensitivity, 100% specificity, 81% NPV, and 100% PPV. The accuracy of IMC was better, compared to culturing (93% vs. 87%). Moreover, the median time to detection was in average 51 h for culturing and only 10 h for IMC [58]. In another study, Chicos et al. have demonstrated that IMC provided similar sensitivity, accuracy and specificity, as standard culturing, using samples collected form patients with fracture-related infections (FRI). However, IMC had a shorter median time to detection (1.5 h vs. 51.5 h) [59].

Treatment of multidrug-resistant bacteria related infection is still a great challenge in clinical practice. Early detection of these bacterial strains is crucial in the management of these infections. In a multicenter study, Tellapragada et al. tested the efficacy of microcalorimetry in determining the MIC (minimum inhibitory concentration) for extensively drug-resistant bacteria strains, such as *Escherichia coli*, *Klebsiella pneumoniae*, *Pseudomonas aeruginosa* and *Acinetobacter baumannii*. Data of a total of 316 isolates were analyzed. They found that IMC could accurately indicate the MIC of different types of antibiotics for the clinical bacteria isolates studied. In their experiments, the endpoint to measure MIC was at 18 h [60].

Biofilm forming bacteria are capable to produce a protective matrix called EPS (extracellular polymeric substance), blocking the penetration of most of the antibiotics to the bacterial colony [61,62]. Important to note, that most of the implant-related infections are caused by biofilm forming bacterial strains. Importantly, isothermal microcalorimetry has the potential to investigate biofilm-related infections [63]. Sultan et al. created a reproducible biofilm-model to use isothermal microcalorimetry to follow the response of biofilm towards antibiotic treatment in real-time. Their calorimetry-based biofilm susceptibility assay demonstrated the high-level of resistance of *Staphylococcus aureus* biofilms to the tested antibiotics [64]. Butini et al. found that after MRSA biofilm treated with 1024 μg/mL vancomycin, a lower percentage (∼5) of cells could survive in a metabolically inactive form (persisters). However, IMC could confirm that ‘persister bacteria’ reverted to a normal-growing phenotype placed in an antibiotic-free environment [65]. To further facilitate the calorimetric diagnosis of biofilm-forming bacteria, Said et al. developed a flow-system instead of the use of biofilms in standard closed ampoules, which system could be built into a microcalorimeter [66].

The reliability of standard microbiological culturing, multiplex PRC (polymerase chain reaction) and isothermal microcalorimetry test were compared in the diagnostics of septic arthritis. The standard culturing technique has its limitations: time and sensitivity, especially in conditions when antibiotics are administered prior to sampling. Broad range, 16S ribosomal DNA PCR has limited sensitivity and is a challenge to determine mixed bacterial infections. Similarly, multiplex PCR has its limitations based on type of primers containing the cartridge. Morgenstern et al. analyzed SF (synovial fluid) samples from patients with acute arthritis [67]. In the septic cases, SF leukocyte count and granulocyte percentage indicated the highest sensitivity (94%), both standard cultures and microcalorimetry showed positive results in 46%, while multiplex PCR was positive only in 23% of the cases. Meanwhile, the accuracy of SF culture, PCR, and microcalorimetry were similar.

**Table 2:** Application of thermal analysis in the diagnostics of septic conditions.

Disease/Tested Samples	Method	Publications	Result Summary
*Escherichia coli* cultures in TSB (tryptic soy broth)	Differential scanning calorimetry (DSC)	Rangel-Vargas et al. [47].	During the initial growth phase, bacteria released energy and the thermograms produced by their measurements correlated to the cell growth and activity, supported by plate counts. DSC detected the real time growth of *E. coli*, between few minutes up to 5 h.
Broth cultures	Micro-DCS (microcalorimetry) Isothermal microcalorimetry (IMC)	Muntean et al. [48].	Thermograms were recorded from broth cultures of Escherichia coli (ATCC 25922) and Staphylococcus aureus (ATCC 25923) at an initial 1/100 dilution of 0.1 McFarland suspension. They claimed that the thermograms behaved as a kinetic fingerprint;
Venous catheter infections, wild-type bacteria (*Escherichia coli*, *Staphylococcus epidermidis*, *Klebsiella pneumoniae*, and *Enterococcus faecium*) collected from blood cultures	Isothermal microcalorimetry (IMC)	Austin et al. [49].	Isothermal microcalorimetry confirmed the bacterial infection thermograms species-specific pattern, with highly reproducible data within each species (within-batch %CV usually about ≤1%).
Urine infection	Isothermal microcalorimetry (IMC)	Braissant et al. [50].	The assessment of bacterial growth in artificial urine samples inoculated with the most common UTI pathogens: *Escherichia coli*, *Enterococcus faecalis* and *Enterococcus faecium*).
Urine infection	Isothermal microcalorimetry (IMC)	Bonkat et al. [51]	Sterile urine samples were inoculated with four different kinds of bacterial strains: *Escherichia coli*, *Proteus mirabilis*, *Enterococcus faecalis* and *Staphylococcus aureus*.
Bacterial over infection of human synovial samples	DSC in isothermal mode	Dandé et al. [33,37,52]	*Staphylococcus aureus*, MRSA (methicillin-resistant *Staphylococcus aureus*), *Staphylococcus epidermidis*, *Streptococcus pyogenes*, *Escherichia coli* and *Pseudomonas aeruginosa*, with colony-forming units (CFU) 10^3^ or 10^5^ colonies/mL broth. Thermal scans were dependent on bacterial concentration, and the scans showed a unique pattern as a characteristic of different bacterial strains.
PJI, isolation of biofilm forming bacteria	Sonication combined with isothermal microcalorimetry (IMC)	Trampuz et al. [54]	They demonstrated that the sensitivity and specificity of microcalorimetry of sonication fluid were 100% and 97%, respectively.
PJI, clinical isolates from bone infections	Isothermal microcalorimetry (IMC)	Christensen et al. [55]	They demonstrated that IMC could detect bacteria within 4 h and determine antimicrobial susceptibility profiles within 2–22 h (median 4.85, range 1.28–21.78). A decision tree, based on antibiotic susceptibility, accurately categorized pathogens, achieving high accuracy (74–100%), sensitivity (100%), and specificity (65–100%).
PJI, synovial samples by aspiration prior to revision of hip and knee surgery	Isothermal microcalorimetry (IMC)	Morgenstern et al. [22].	In 98 of 107 patients (92%), synovial fluid culture and microcalorimetry indicated concordant results (38 PJI and 60 aseptic cases).
PJI, synovial fluid samples	Isothermal microcalorimetry (IMC)	De Bleeckere et al. [56]	Conventional culturing (CC) technique vs. culturing in synthetic synovial fluid (SSF2) or using isothermal microcalorimetry (IMC). Culturing in SSF2: highest positivity rate (53.2 %), followed by IMC and CC (34.2 % and 32.9 %, respectively). Median detection time with IMC: between 15.6 h and 15.9, depending on different culturing media
PJI, tissue samples of patients underwent revision hip or knee arthroplasties	Isothermal microcalorimetry (IMC)	Chicos et al. [58].	Study involved 152 patients with 592 tissue samples. Found concordance between standard microbiological culturing and IMC of 90%. IMC showed a sensitivity of 83%, specificity of 100%, negative predictive value (NPV) of 89%, and positive predictive value (PPV) of 100% for PJI vs. standard culturing showed 69% sensitivity, 100% specificity, 81% NPV, and 100% PPV. The accuracy of IMC was better, compared to culturing (93% vs. 87%). The median time to detection was in average 51 h for culturing vs. 10 h for IMC.
Samples collected form patients with fracture-related infections (FRI)	Isothermal microcalorimetry (IMC)	Chicos et al. [59]	IMC provided similar sensitivity, accuracy and specificity, as standard culturing, using samples collected form patients with fracture-related infections (FRI). IMC had a shorter median time to detection (1.5 h vs. 51.5 h).
Multicenter study, extensively drug-resistant bacteria strains, testing clinical isolates	Isothermal microcalorimetry (IMC)	Tellapragada et al. [60].	Tested the efficacy of microcalorimetry in determining the MIC (minimum inhibitory concentration) for extensive drug-resistant bacteria strains, such as *Escherichia coli*, *Klebsiella pneumoniae*, *Pseudomonas aeruginosa* and *Acinetobacter baumannii*. Data of a total of 316 isolates were analyzed. IMC could accurately indicate the MIC and the endpoint to measure MIC was at 18 h.
Reproducible biofilm-model, *Staphylococcus aureus* infected biofilm	Isothermal microcalorimetry (IMC), calorimetry-based biofilm susceptibility assay	Sultan et al. [64].	Biofilms containing 5 methicillin-sensitive *S. aureus* (MSSA) and 5 methicillin-resistant *S. aureus* (MRSA) strains from different genetic lineages were tested. Their calorimetry-based biofilm susceptibility assay demonstrated the high-level of resistance of *Staphylococcus aureus* biofilms to the tested antibiotics.
Reproducible biofilm-model, *Staphylococcus aureus* infected biofilm	Isothermal microcalorimetry (IMC), calorimetry-based biofilm susceptibility assay	Butini et al. [65].	MRSA biofilm treated with 1024 μg/mL vancomycin, a lower percentage (∼5) of cells could survive in a metabolically inactive form (persisters). IMC confirmed that ‘persister bacteria’ reverted to a normal-growing phenotype placed in an antibiotic-free environment.
*Staphylococcus aureus* biofilms	Isothermal microcalorimetry (IMC), flow-system combined with microcalorimeter	Said et al. [66]	To further facilitate the calorimetric diagnosis of biofilm-forming bacteria, developed a flow-system instead of the use of biofilms in standard closed ampoules, which system could be built into a microcalorimeter.
Synovial samples, collected from patients with acute arthritis	Isothermal microcalorimetry (IMC)	Morgenstern et al. [67].	In the septic cases, SF leukocyte count and granulocyte percentage indicated the highest sensitivity (94%), both standard cultures and microcalorimetry showed positive results in 46%, while multiplex PCR was positive only in 23% of the cases. The accuracy of SF culture, PCR, and microcalorimetry were similar.

## 5. Application of Thermal Analysis in the Diagnostics of Other Diseases

Using human plasma samples, differential scanning calorimetry has been introduced as a potential tool in the diagnostics of malignant diseases two decades ago. Pioneering in this field, Nichola C. Garbett started to promote DSC for detection of changes in unfractionated plasma proteome [17,20] (Table 3). Since then, thermal analyses have been successfully used in the diagnostics of a variety of malignant and inflammatory diseases [18,19,68,69,70,71,72]. The advantage of the sampling for the method, called recently ‘thermal liquid biopsies (TLB)’, is that a relatively small volume of fluid (aspirate, synovia, blood or serum) is sufficient for calorimetric measurements. Recently, Reger et al. introduced automated baseline-correction and signal-detection algorithms with web-based implementation to address certain limitations of the wider use of DSC in the clinical settings [73].

Thermal liquid biopsies showed a distinctive thermal profile in the different types of myocardial injuries. The authors concluded that TLB could be a useful tool in the investigation of cardiovascular diseases [74]. Furthermore, Ferencz et al. have found that the extent of surgical interventions and the consequential stress have a significant impact on the blood plasma thermodynamic parameters [75].

The effect of deep-freezing on different types of tissue has also been investigated by differential scanning calorimetry [76,77]. DSC has also been found useful in the investigation of different drug effects and pharmaceutical intervention [78]. In another study, Gaszler et al. have compared the thermal parameters of red blood cells (RBCs) harvested from diabetic and non-diabetic patients [79].

**Table 3:** Application of thermal analysis in the diagnostics of other diseases.

Disease/Tested Samples	Method	Publications	Result Summary
Malignant diseases, TLB	Differential scanning calorimetry (DSC)	Garbett et al. [17,18,19,20]	Detection of changes in unfractionated plasma proteome in several types of malignant diseases, analyzed 16 most abundant purified plasma proteins.
Acute thrombotic myocardial infarction (TMI), acute non-thrombotic myocardial injury (nTMi), and chronic coronary atherosclerotic disease (cCAD), TLB	Differential scanning calorimetry (DSC)	Lidani et al. [74].	Distinctive thermal signatures were found in the median TLB profiles across the three patient types.
Surgery patients, TLB: Plasma	Differential scanning calorimetry (DSC)	Ferencz et al. [75,80]	The extent of surgical interventions and consequential stress have a significant impact on the blood plasma thermodynamic parameters.
Peripheral arterial disease, cilostazol treated patients; TLB: Plasma and serum	Differential scanning calorimetry (DSC)	Benkő et al. [78]	Plasma analysis may provide a more comprehensive and sensitive representation of drug-induced changes in blood properties, compared to the serum.
Red blood cells (RBCs) harvested from diabetic and non-diabetic patients	Differential scanning calorimetry (DSC)	Gaszler et al. [79]	Diabetes caused atypical cell morphology of RBCs and in a case-dependent manner, increased thermal stability of cytoplasm and decreased thermal stability of the cell membrane.

## 6. Potential Limitations

One of the potential limitations of several earlier studies was that IMC could confirm the presence of bacteria based on heat production, but its usefulness in the identification of specific bacteria in clinical settings had not been fully supported. Therefore, Lozano-García et al. tested the automatic identification of PJI causing bacteria, analyzing heat flow curves by machine learning and transfer learning CNN (convolutional neural network) models [81]. For instance, they achieved 100% accuracy in PJI detection, using a binary XGBoost classifier. Their data suggest that IMC with machine learning modules could be used in the clinical setting to identify PJI pathogens.

Despite the number of recent publications, there is only limited evidence available to support and justify the routine application in clinical settings. Most of the studies show only small sample sizes and use single-center designs. Many studies lack external validation. In other cases, instead of collecting urine samples from UTI patients or synovial samples from patients with septic or rheumatoid arthritis, artificial bacterial inoculations were utilized, adding bacterial strains to sterile urine samples or synovial fluids.

As Zalavras has written in the commentary, further investigations are needed to address important questions in regard to the application of isothermal microcalorimetry in the clinical routine. While the use of isothermal microcalorimetry could reduce the time to diagnosis from 2 days to 2 h, it is still too long for an intraoperative diagnosis. The author also suggests replicated studies with larger samples size and longer follow-up time, especially in cases of FRI (fracture-related infections). Zalavras emphasises the need to further determine the ability of isothermal microcalorimetry to produce bacterial specification and sensitivity [82].

Most available studies would qualify only as proof-of-concept (PoC), representing the first phase of translational research. Therefore, it is needed to be emphasized that despite the promising data of Lozano-García et al., most authors have tested the method on their own sample collections, and multicentric studies are warranted to provide further data for standardization and for testing reproducibility prior to routine clinical application.

Besides the relatively low n-values in the rest of the publications, there are other potential factors that should be considered affecting calorimetric results. DSC and TLB profiles can be influenced by protein composition, systemic disease, medication, storage, and freeze–thaw cycles. IMC can also be affected by bacterial concentration, culture conditions, previous antibiotics, biofilms, polymicrobial infection, and slow-growing organisms [83].

While IMC could be a promising additional diagnostic method in periprosthetic joint infections, it will have to compete with other emerging diagnostic techniques: quantitative polymerase chain reaction (qPCR), multiplex PCR assays and next-generation sequencing (NGS) platforms [84,85]. The use of Scattered Light Integrating Collector (SLIC) technology with tissue samples could also be advantageous, reducing the time to reach diagnosis [86]. Novel, highly accurate biomarkers are available to analyze synovial fluid or biofilm samples [87]. The application of artificial intelligence (AI) also has great potential in the prevention, diagnosis and management of PJI [88].

Lin et al. have analyzed the heat denaturation curves (HDCs) and proteomic profiles of serum proteins of patients with joint-inflammatory symptoms or specifically with rheumatoid arthritis. Besides evaluating the differences in the thermal parameters, they utilized mass spectrometry to assess the proteomic profiles, demonstrating that the differences in the heat denaturation curves were not directly related to differences in the concentration of abundant serum proteins. Instead, the variations in the HDCs can be explained by the altered thermal stability of certain fractions of the sample’s human serum albumin proteins. The authors claimed that the shifts in the melting temperature of serum proteins in RA patients could be caused by changes in the ligand- or protein-binding abilities [41].

On the other hand, the authors suggest that the potential cost and availability of calorimetric analyses should not be a limiting factor, since the measurements could be performed in routine diagnostic laboratories following proper training and, in the future, with the help of automatization.

Naturally, more clinical studies and higher levels of evidence are needed to support the use of the method in the routine human diagnostic protocols of septic complications [55].

**Sources:** Computerized searches of PubMed–National Library of Medicine, Web of Science, and Google Scholar have been performed to find relevant articles. Search terms applied: ‘calorimetry’ combined with ‘fracture related infection’, ‘periprosthetic joint infection’, ‘sepsis’ and ‘inflammation’. The reference lists of all articles collected were examined for more relevant references.

## 7. Conclusions and Outlook

The calorimetric examination of tissue and fluid samples of various origins could be a useful complementary diagnostic tool in the clinical investigation of inflammatory and septic conditions. However, additional research with larger sample sizes is needed to further support the cost effectiveness and reliability of the method.

## Data Availability

No new data were created or analyzed in this study. Data sharing is not applicable to this article.
